# Advancements in nuclear imaging using radiolabeled nanobody tracers to support cancer immunotherapy

**DOI:** 10.1093/immadv/ltae006

**Published:** 2024-08-26

**Authors:** Katty Zeven, Yoline Lauwers, Lynn De Mey, Jens M Debacker, Tessa De Pauw, Timo W M De Groof, Nick Devoogdt

**Affiliations:** Molecular Imaging and Therapy Research Group, Vrije Universiteit Brussel (VUB), Brussels, Belgium; Molecular Imaging and Therapy Research Group, Vrije Universiteit Brussel (VUB), Brussels, Belgium; Molecular Imaging and Therapy Research Group, Vrije Universiteit Brussel (VUB), Brussels, Belgium; Nuclear Medicine Department, UZ Brussel, Brussels, Belgium; Molecular Imaging and Therapy Research Group, Vrije Universiteit Brussel (VUB), Brussels, Belgium; Nuclear Medicine Department, UZ Brussel, Brussels, Belgium; Molecular Imaging and Therapy Research Group, Vrije Universiteit Brussel (VUB), Brussels, Belgium; Molecular Imaging and Therapy Research Group, Vrije Universiteit Brussel (VUB), Brussels, Belgium; Molecular Imaging and Therapy Research Group, Vrije Universiteit Brussel (VUB), Brussels, Belgium

**Keywords:** nuclear imaging, nanobodies, immunotherapy, diagnostics, cancer

## Abstract

The evolving landscape of cancer immunotherapy has revolutionized cancer treatment. However, the dynamic tumor microenvironment has led to variable clinical outcomes, indicating a need for predictive biomarkers. Noninvasive nuclear imaging, using radiolabeled modalities, has aided in patient selection and monitoring of their treatment response. This approach holds promise for improving diagnostic accuracy, providing a more personalized treatment regimen, and enhancing the clinical response. Nanobodies or single-domain antibodies, derived from camelid heavy-chain antibodies, allow early timepoint detection of targets with high target-to-background ratios. To date, a plethora of nanobodies have been developed for nuclear imaging of tumor-specific antigens, immune checkpoints, and immune cells, both at a preclinical and clinical level. This review comprehensively outlines the recent advancements in nanobody-based nuclear imaging, both on preclinical and clinical levels. Additionally, the impact and expected future advancements on the use of nanobody-based radiopharmaceuticals in supporting cancer diagnosis and treatment follow-up are discussed.

## Introduction

Over the past decades, a plethora of cancer immunotherapies have been developed. These include immune checkpoint inhibitors (ICIs) [1], adoptive cell transfer [2], cancer vaccines [3], cytokines [4], antibodies(-derivatives), and antibody-drug conjugates (ADCs) [5, 6]. While many are still in (pre-)clinical testing, several immunotherapies have gained clinical approval to treat certain malignancies [7].

Despite this success, only a small fraction of patients demonstrate a favorable immunotherapy response [8]. This limited efficacy is mediated by the inherent complexities and interplay between tumors and the immune system. To optimize cancer management, a comprehensive understanding of the molecular characteristics (i.e. cancer-specific targets) of both primary and secondary lesions is indispensable to predict or follow-up treatment responses [9]. Besides tumor-specific antigens, numerous factors within the tumor microenvironment (TME) contribute to cancer prognosis and therapy outcome and are employed as biomarkers [10, 11].

Currently, molecular characterization of tumor or TME-specific targets is performed primarily via histological analysis of biopsies, complemented by screening of circulating cancer markers when applicable [12]. However, these methods do not provide whole-body spatiotemporal information, as tumor lesions are often inaccessible, the TME is dynamic, and intra- and inter-tumoral heterogeneity exists among many cancer types [12]. To address these challenges, noninvasive imaging of tumor or TME-specific targets allows for whole-body visualization. This approach reveals spatiotemporal changes in these markers, facilitating the monitoring of immunotherapy responses and potentially advancing personalized treatment strategies [13].

A radionuclide-based imaging tracer typically consists of two components: a radionuclide coupled to a targeting moiety. These tracers are visualized using medical imaging modalities such as positron emission tomography (PET) and single photon emission computed tomography (SPECT) [14]. These modalities offer good spatial resolution and allow for the quantitative evaluation of tracer signals. Various radionuclides are available for PET and SPECT imaging. For PET imaging, commonly used radionuclides include ^68^Ga, ^18^F, ^64^Cu, and ^89^Zr. For SPECT imaging, ^99m^Tc, ^111^In, and ^131^I are commonly used (Table 1) [14].

**Table 1.** Overview of radionuclides commonly used for PET and SPECT imaging and TRT of Nbs.

Radionuclide	Half-life (t_1/2_)	Commonly used chelators or prosthetic groups	Decay mode	SPECT/PET/TRT
Gallium-68 (^68^Ga)	68 min	NOTA/DOTA/NODAGA/THP	ε+β^+^ (100%)	PET
Fluorine-18 (^18^F)	110 min	SFB/FPy-TFP NOTA/RESCA (AI-^18^F)	β^+^ (100%)	PET
Copper-64 (^64^Cu)	13 h	NOTA/DOTA/NODAGA/DTPA	ε+β^+^ (61.5%), β^-^ (38.5%)	PET
Zirconium-89 (^89^Zr)	4 days	DFO/DFO*	ε+β^+^ (100%)	PET
Technetium-99m (^99m^Tc)	6 h	NA Tricarbonyl chemistry	IT	SPECT
Indium-111 (^111^In)	3 days	DOTA/DTPA	ε (100%)	SPECT
Iodine-131 (^131^I)	8 days	SGMIB	β^-^ (100%)	SPECT/TRT
Bismuth-213 (^213^Bi)	46 min	DOTA/DTPA	β^-^ (37.9%), α (2.1%)	TRT
Lutetium-177 (^177^Lu)	7 days	DOTA/DTPA	β^-^ (100%)	SPECT/TRT
Terbium-161 (^161^Tb)	7 days	DOTA/DTPA	β^-^ (100%)	SPECT/TRT
Actinium-225 (^225^Ac)	10 days	DOTA	α (100%)	TRT

α: alpha decay; β^+^: beta plus decay; β-: beta minus decay; DFO: desferrioxamine; DOTA: 1,4,7,10-tetraazacyclododecane-1,4,7,10-tetraacetic acid; DTPA: diethylenetriaminepentaacetic acid; ε: electron capture; Fpy-TFP: 6-[^18^ F]fluoronicotinyl-2,3,5,6-tetrafluorophenyl ester; [^18^F]SFB: N-succinimidyl-4-[^18^F]fluorobenzoate; IT: Isomeric transition; NODAGA: 1,4,7-triazacyclononane,1-glutaric acid-4,7-acetic acid; NOTA: 1,4,7-Triazacyclononane-1,4,7-triacetic acid; PEG: polyethylene glycol; PET: positron emission tomography; SGMIB: N-succinimidyl 4-guanidino-methyl-3-iodobenzoate; SPECT: single photon emission computed tomography; THP: tris(hydroxypyridinone; TRT: targeted-radionuclide therapies.

**Figure 1.**
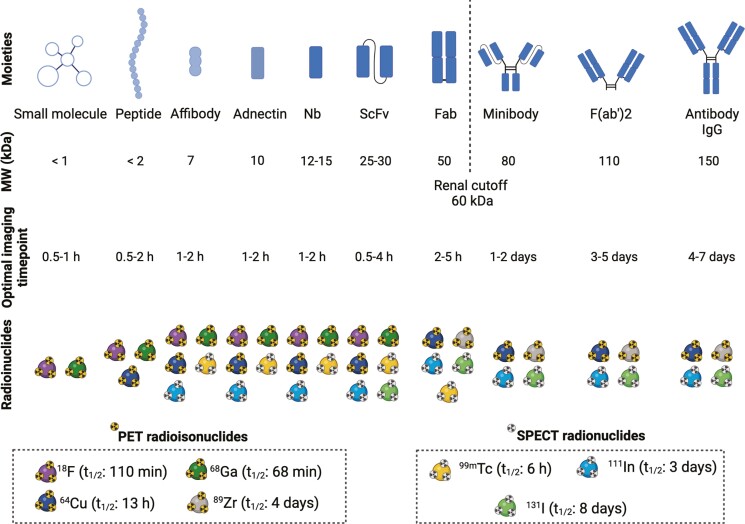
Overview of different targeting moieties for PET and SPECT imaging. Their unique characteristics (molecular weight and optimal imaging timepoint) and matching radionuclides are presented. ^64^Cu: copper-64; ^18^F: fluorine-18; Fab: antigen-binding fragment; ^68^Ga: gallium-68; ^111^In: indium-111; ^131^I: iodine-131; MW: molecular weight; Nb: nanobody; PET: positron emission tomography; ScFv: single-chain variable fragment; SPECT: single-photon emission computerized tomography; ^99m^Tc: technetium-99m; ^89^Zr: zirconium-89. Created with BioRender.com.

Moreover, numerous targeting moieties have been developed, each with specific characteristics. These include small molecules, peptides, scaffold proteins, antibody fragments, and monoclonal antibodies (mAbs) (Fig. 1). Numerous clinical trials are currently investigating mAb-based tracers to predict immune therapy responses. Additionally, radiolabeled mAbs have gained significant attention as therapeutic compounds in targeted-radionuclide therapies (TRT). For example, ^90^Y-labeled ibritumomab targeting CD20 for the treatment of B-cell lymphoma [15]. TRT enables the systemic delivery of cytotoxic radiation directly to the target, minimizing damage to surrounding cells and tissues. Other examples of therapeutic radionuclides applied in TRT are ^131^I, ^213^Bi, ^177^Lu, ^161^Tb, and ^225^Ac (Table 1) [15]. The combination of diagnostics and (radio)therapy has led to a new field known as (radio)theranostics, which aims to achieve controlled and personalized drug delivery while simultaneously visualizing therapy response through molecular imaging [15].

However, the substantial size of mAbs (~150 kDa) and the presence of an Fc-domain inherently limit their tissue penetration capacity and extend their blood circulation time. Consequently, these characteristics result in low target-to-background ratios and low-contrast images during early imaging timepoints. As a result, imaging with mAb-based tracers typically occurs several days post-injection, necessitating the use of longer-lived radioisotopes such as ^89^Zr. This leads to an increased radiotoxicity compared to short-lived radioisotopes. Furthermore, the interval between tracer administration and imaging presents logistical challenges for patients and clinical practice. Moreover, the slow pharmacokinetics of mAbs are also suboptimal for the safety profile of TRT [16]. Consequently, smaller antibody fragments with more favorable characteristics for radiotheranostics are being explored.

Single domain antibodies (sdAbs) or nanobodies (Nbs), derived from heavy-chain-only antibodies of camelids, have emerged as promising imaging tools over the last decade [17]. Nbs are smaller compared to mAbs (12–15 kDa), yet share similar traits of high stability and solubility, while having better tissue and tumor penetration [18]. Typically, Nbs are generated after immunization of camelids with specific target antigens from certain species. Next, B lymphocytes are isolated to create an immune library. From this library, genes encoding antigen-specific Nbs are selected through several rounds of phage display, and antigen-specific Nbs are subsequently produced recombinantly in *E. coli* strains for further characterization and selection. This method, along with other methods used for generating Nb libraries, is extensively described in a review of Muyldermans [19]. Despite their camelid origin, Nbs demonstrate high homology with human antibody heavy chain variable domains, thereby minimizing their immunogenicity [20]. Moreover, when a high homology between the human and mouse sequences of a certain target exists, cross-reactive Nbs can be created, facilitating more straightforward clinical translation [21, 22]. Furthermore, Nbs undergo rapid clearance from the blood because their size is far below the renal clearance cut-off of ~60 kDa. These fast pharmacokinetics result in high tumor-to-background ratios as early as 1 h after tracer injection, significantly enhancing their diagnostic and therapeutic applications (e.g. TRT) compared to mAbs [23]. This rapid clearance of Nbs allows for the use of short-lived radionuclides such as ^68^Ga, and ^18^F for PET imaging and ^99m^Tc for SPECT imaging [24, 25]. Additionally, short-lived radionuclides facilitate repeated imaging, which is ideal for monitoring therapeutic responses. However, in some cases, the rapid clearance of Nbs can result in lower tracer uptake. To address this, polyethylene glycol (PEG)ylation has been described in a few preclinical studies, where the conjugation to a 20 kDa PEG-moiety results in a longer serum half-life, leading to increased tracer uptake and improved imaging signals [26]. However, PEGylation increases the apparent size of the tracer, necessitating the use of longer-lived radionuclides. This ultimately results in a higher radiation dose, making it less favorable.

The selection of a radiolabeling method depends on the targeting moiety and the specific radionuclide being used. Radionuclides can be classified into two categories: radiometals (e.g. ^68^Ga, ^64^Cu) and radiohalogens (e.g. ^18^F, ^131^I). For radiometals, chelators such as NOTA, DOTA, DFO, and NODAGA are commonly used to form stable complexes with radionuclides [27, 28]. The selection of a chelator depends on the choice of radiometal, where characteristics such as half-life as well as the characteristic of the targeting moiety, such as (thermo)stability, functionalization strategies in case of biomolecules, impact the stability of the radiometal-chelator complex and prevent *in vivo* demetallation or transchelation.

Furthermore, radiolabeling can be either direct (single-step) or indirect (two-step using prosthetic groups). For instance, Nbs with a hexahistidine-tag can be directly labeled with ^99m^Tc-tricarbonyl. In contrast, labeling Nbs with ^18^F requires the use of a prosthetic group such as succinimidyl-4-[^18^F]fluorobenzoate ([^18^F]SFB) because direct labeling with ^18^F requires harsh conditions that may damage the Nb’s functionality [29, 30]. The exception here is AI^18^F-RESCA, which allows for direct radiofluorination of heat-sensitive molecules [31]. Finally, radiolabeling of biomolecules with both radiometals or radiohalogens can be performed using random or site-specific functionalization strategies, each with its advantages and disadvantages. The most straightforward strategy is random labeling, whereby the radionuclide is linked to naturally occurring amino acids (such as lysines) from the targeting moieties. However, this results in a heterogeneous product with possibly varying pharmacokinetic properties limiting the possibility to perform side-by-side comparisons. In contrast, site-specific strategies (such as Sortase-A or maleimide-thiol mediated conjugation) introduce the radionuclide to a single and specific site to form a uniform product, improving reproducibility and maintaining the tracer’s affinity [32, 33]. However, site-specific labeling often requires engineering an extra tag, which can influence the production process [33]. Additionally, site-specific labeling can increase the production costs due to added components and purification steps compared to the random strategy.

Overall, the favorable properties of Nbs position them as attractive alternatives for radionuclide-based imaging. Several Nb-based imaging tracers have been evaluated (pre-)clinically with encouraging results.

This review provides a comprehensive overview of the recent advancements in Nb-based tracers tailored for human (h) and/or mouse (m) tumor-specific or TME-specific targets. Additionally, the impact and expected future advancements of noninvasive and nuclear imaging techniques in supporting cancer diagnosis and treatment follow-up are discussed.

## Nanobody-based imaging of tumor-specific antigens

Many Nb-based tracers have been extensively investigated to visualize and molecularly characterize cancerous lesions for better diagnosis and therapy prediction, but only a few have progressed to clinical evaluation (Table 2). Some Nbs also demonstrated radiotheranostic potential when labeled with a therapeutic radionuclide. These include Nbs directed to cancer-specific antigens, the tumor stroma, the extracellular matrix (ECM), or the neo-vasculature. For example, Nbs against prostate-specific membrane antigen (PSMA) for targeting prostate cancer, fibroblast activation protein (FAP) to target cancer-associated fibroblasts or insulin-like growth factor binding protein-7 for the visualization of the neo-vasculature have been described [34]. In this review, we will highlight the human epidermal growth factor 2 (HER2) as a tumor-specific target. More information on different anti-cancer Nbs and their specifications can be found in another review [17].

**Table 2.** Radiolabeled nanobodies to image tumor-specific antigens under clinical investigation.

Target	Reactivity	Clone	Radionuclide and chelator	Cancer type	Discovery stage	Reference
HER2	Human	2Rs15d	^99m^Tc, ^68^Ga-NOTA	HER2+ breast cancer	Clinical Phase I	[36, 37, 44]
				Breast cancer and HER2+ solid tumors	Clinical Phase II	[38] NCT03924466
				Brain-metastasized solid tumors	Clinical Phase II	NCT03331601
		NM-02	^99m^Tc	Breast cancer	Clinical Phase I	[39] NCT04040686
				Breast cancer	Clinical Phase I	[41] NCT04674722
		MIRC208	^99m^Tc	HER2+ cancer	Clinical Phase I	[42] NCT04591652
		MIRC213	^99m^Tc	HER2+ breast cancer	Clinical Phase I	NCT05622240
			[^18^F]AlF (RESCA)	HER2+ cancer	First-in-human	[32]
CEA	Human	HNI01	^68^Ga-THP	Colorectal cancer	Clinical Phase I	[45]
Trop2	Human	Trop2 Nb	^68^Ga-THP	Solid tumors	Clinical Phase I	NCT06188468
		T4	^68^Ga-NOTA	Solid tumors	Clinical Phase I	[46] NCT06203574
CLDN18.2	Human	ACN376	^68^Ga	Solid tumors	Clinical Phase I	[47] NCT05436093
CD38	Human	NB381	^68^Ga	Multiple myeloma	Clinical Phase I	NCT06385652

CEA: Carcinoembryonic antigen; CLDN18.2: Claudin18.2; ^18^F: fluorine-18; [^18^F]AlF: aluminum-[^18^F]fluoride; ^68^Ga: gallium-68; HER2: human epidermal growth factor 2; NOTA: 1,4,7-Triazacyclononane-1,4,7-triacetic acid; ^99m^Tc: technetium-99m; THP: tris(hydroxypyridinone; Trop2: Tumor-associated calcium signal transducer 2.

### Human epidermal growth factor 2

Among the ERBB/HER transmembrane tyrosine kinase receptor family, HER2 stands out as one of the most extensively studied cancer markers. HER2 is a key therapeutic target for breast cancer, although its overexpression is also observed in other malignancies, such as gastric and ovarian cancer. While HER2-overexpression is a known negative prognosticator in breast cancer patients, the introduction of the HER2-inhibiting mAbs (e.g. trastuzumab) has significantly changed the treatment regimen [35]. Initially, the therapeutic effects of HER2-targeting mAbs were demonstrated in lesions with high HER2-overexpression. However, novel HER2-targeting therapies, including ADCs and small-molecule tyrosine kinase inhibitors, have also shown therapeutic effects in patients with low HER2 expression [35].

**Figure 2.**
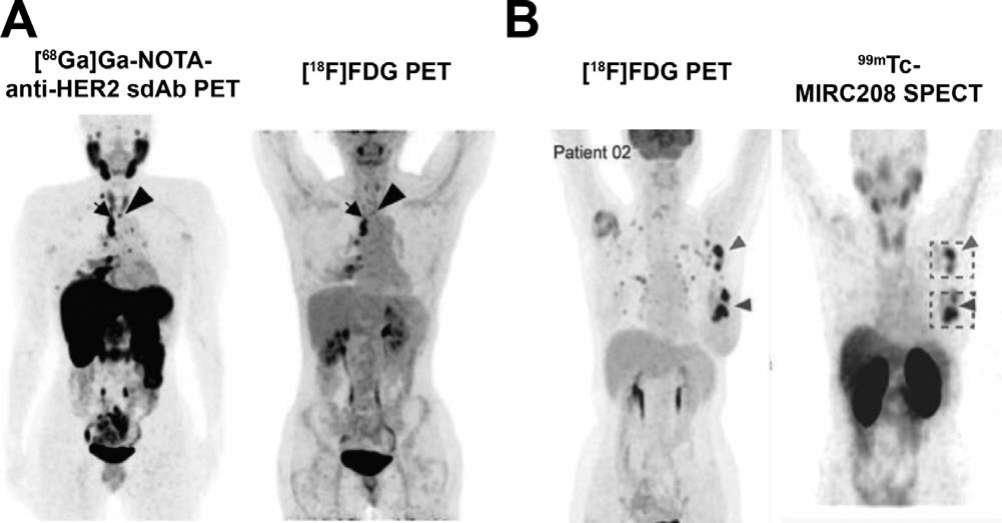
Examples of the clinical applicability of nanobody-based nuclear imaging of human epidermal growth factor receptor 2. (A) [^68^Ga]Ga-NOTA-anti-HER2-sdAb (left) and [^18^F]FDG (right) maximum-intensity projection PET images of a patient with a HER2-positive (3+) invasive ductal breast carcinoma with [^18^F]FDG-avid lymph nodes in the mediastinum. The extent of disease to the cervical lymph nodes (arrow) was better delineated on [^68^Ga]Ga-NOTA-anti-HER2-Nb as compared to [^18^F]FDG PET. (B) Representative maximum-intensity projection images of [^18^F]FDG PET (left) and ^99m^Tc-MIRC208 SPECT (right) in a patient with HER2 overexpression (3+). [^18^F]FDG: ^18^F-2-fluoro-2-deoxyglucose; ^68^Ga: gallium-68; Nb: nanobody; PET: positron emission tomography; SPECT: single-photon emission computerized tomography; ^99m^Tc: techenetium-99m. Images have previously been published in an adapted form by: (A) JNM. Gondry *et al*. Phase II Trial Assessing the Repeatability and Tumor Uptake of [^68^Ga]Ga-HER2 Single-Domain Antibody PET/CT in Patients with Breast Carcinoma. J Nucl Med. 2024; 65(2):178-184. © SNMMI [38]; and (B) Theranostics. Liqiang *et al*. HER2-targeted dual radiotracer approach with clinical potential for noninvasive imaging of trastuzumab-resistance caused by epitope masking. Theranostics. 2022; 12(12):5551-5563 [42], under a CC BY 4.0 license.

To enable patient stratification for HER2-targeting therapies, several research groups have developed Nbs against HER2 for noninvasive SPECT or PET imaging of the spatiotemporal HER2 expression, and which are currently undergoing clinical testing. In 2011, the Nb 2Rs15d labeled with ^99m^Tc was first described, showing high uptake in two HER2^+^ mouse tumor models at 1 h post-injection, while binding a distinct epitope from the anti-HER2 therapeutic mAbs trastuzumab and pertuzumab [36]. Currently, 2Rs15d is being evaluated clinically for HER2 PET imaging. Phase I results were published in 2016, with no AEs (2 h post-injection) and no detection of anti-drug antibodies (ADAs) up until three months after administering three different doses of 2Rs15d [37]. Moreover, tracer uptake in primary breast cancer lesions was visible in 13 out of 15 patients, with high accumulation also observed in the kidneys, liver, intestines, and HER2^+^ metastases at 1–1.5 h post-injection [37]. In a subsequent phase II study (n=20), the tracer showed its ability to assess intratumoral HER2 heterogeneity more effectively than [^18^F]FDG, proving its potential to follow-up HER2-targeted therapy response in the future (Fig. 2) [38]. Two additional phase II studies are currently ongoing to evaluate the tracer in non-breast tumors and breast cancer patients undergoing neoadjuvant therapy (NCT03924466), as well as in HER2^+^ brain metastases (NCT03331601).

Besides 2Rs15d, three other HER2-targeting Nbs have undergone clinical translation to test their efficacy in diagnosing and monitoring HER2+ cancers undergoing targeted therapies: NM-02, MIRC208, and MIRC213. The phase I clinical trial with ^99m^Tc-NM-02 displayed the tracers’ safety and potential to identify HER2 positivity [39]. A follow-up study using ^99m^Tc- and Rhenium-188 (^188^Re, *t*_1/2_ = 17 h)-labeled NM02 for SPECT/CT and TRT, respectively, is ongoing (NCT04674722). A study with ^131^I-NM-02 also demonstrated a radiotheranostic potential [40]. The first results from the SPECT/CT evaluation with ^99m^Tc-NM-02 revealed a positive correlation between uptake and HER2 expression in the untreated group (*n* = 24) while also showing a preliminary potential for the tracer to monitor the therapeutic effects of HER2-targeted therapy [41].

MIRC208 and MIRC213, target distinct epitopes of the HER2 protein with the respective purpose of patient selection and HER2 accessibility [42]. Both Nbs, labeled with ^99m^Tc for SPECT imaging, are currently undergoing phase I trials. Initial results from two patients injected with ^99m^Tc-MIRC208 demonstrated its preliminary toxicity profile and lesion accumulation (Fig. 2) (NCT04591652) [42]. Notably, a RESCA-coupled version of MIRC213 has recently been labeled with ^18^F for PET imaging, demonstrating its safety and proof-of-concept in six patients [32].

In addition to HER2-targeting Nbs, it is important to note that other HER2-targeting tracers, including mAbs, antibody derivatives, scaffold proteins, and peptides have demonstrated potential for patient stratification and therapy monitoring in the clinic. These tracers have been described in another review [43].

## Nanobody-based imaging of the tumor microenvironment

### Nanobody-based imaging of immune checkpoints

Immune checkpoints (ICs) are critical regulators of immune responses, maintaining homeostasis and preventing collateral damage. However, ICs can be exploited within the TME to induce an immune-suppressive environment, hindering anti-cancer immune responses [48]. Examples include cytotoxic T-lymphocyte-associated protein 4 (CTLA-4) and programmed death-1 (PD-1) along with one of its ligands (PD-L1). Following this discovery, IC-targeting mAbs have revolutionized immunotherapy [48]. Particularly, ICIs against PD-(L)1 have become the current standard of care for various cancers, including melanoma, head-and-neck, and non-small-cell lung carcinoma (NSCLC). Nevertheless, only a fraction (20–40%) of patients benefit from these therapies, with others either not responding or developing acquired resistance [49]. To address this, next-generation ICIs targeting novel ICs, including Lymphocyte-Activated Gene-3 (LAG-3), T cell receptor with Ig and ITIM domain (TIGIT), and T-cell immunoglobulin and mucin domain-containing protein 3 (TIM-3), have been developed to enlarge the treatment options [50]. Several radiolabeled Nbs specific for ICs have been developed to noninvasively image the dynamic IC expression within the TME and serve as diagnostic companions for patient stratification. Some of these Nbs also showed potent IC blockade capablities, indicating their potential for a theranostic approach (Table 3 and Fig. 4). Moreover, some of these Nbs can also predict ICI therapy response or resistance, as such enhance the outcomes of the treatment. Notably, therapy response prediction has also been demonstrated with other tracer formats such as mAbs, adnectins, and peptides [51].

**Table 3.** Overview of radiolabeled nanobodies in (pre-)clinical stage to image immune-associated targets.

Target	Reactivity	Clone	Radionuclide and chelator	Cancer type	Discovery stage	Reference
PD-L1	Human	K2	^99m^Tc, ^68^Ga	Melanoma cell line, breast cancer cell line	Preclinical	[33, 58]
	Human	Nb109	^68^Ga	Glioblastoma cell line, Melanoma, Patient-derived lung cancer xenografts	Preclinical	[61–63]
	Human	APN09	^68^Ga-NOTA, ^99m^Tc	Lung adenocarcinoma cell line, NSCLC	Preclinical	[64, 92]
			^68^Ga-THP	NSCLC	Clinical Interventional	[64] NCT05156515
	Human	Nb1	^68^Ga	Solid tumors	Clinical Interventional	NCT06383598
	Human	NM-01	^99m^Tc	NSCLC	Clinical Phase II	[65, 93] NCT04436406 & NCT04992715NCT02978196
	Human	RW102	^68^Ga-NOTA	NSCLC	Clinical interventional	[67] NCT06165874
	Mouse	B3, A12	^18^F, ^64^Cu	Brown adipose tissue	Preclinical	[60, 94]
	Mouse	A12	^18^F, ^64^Cu	Brown adipose tissue, Melanoma cell line	Preclinical	[60, 94]
	Mouse	C3, E2, E4, C7	^99m^Tc	Lung epithelial carcinoma cell line	Preclinical	[56]
	Mouse	MY1523	^99m^Tc	Colorectal carcinoma cell line, Breast cancer cell line, Non-Hodgkin lymphoma cell line	Preclinical	[59]
PD-L2	Human	Mirc415	^68^Ga	Solid tumors	Clinical Interventional	NCT05803746
CTLA-4	Mouse	H11	^18^F, ^89^Zr	Melanoma cell line	Preclinical	[26]
LAG-3	Human	3187	^99m^Tc	Lung carcinoma cell line	Preclinical	[70]
	Mouse	3132/3206	^99m^Tc	Lung carcinoma cell line, Colorectal carcinoma cell line	Preclinical	[69, 71]
TIGIT	Human	16925	^99m^Tc	Lung carcinoma cell line	Preclinical	[74]
	Human	Nb138	^68^Ga	Melanoma cell line	Preclinical	[75]
	Mouse	16988	^99m^Tc	Lung carcinoma cell line	Preclinical	[74]
CD70	Human	CD70 VHH	^68^Ga	Renal cell carcinoma cell line, Lung adenocarcinoma cell line	Preclinical	[76]
	Human	RCCB6	^18^F	Renal cell carcinoma	Clinical interventional	NCT06148220 [77]
CD8	Mouse	VHH-X118	^89^Zr	Breast cancer cell line, melanoma cell line, Adenocarcinoma cell line, Pancreatic cancer cell line	Preclinical	[80, 81]
	Human/Monkey	SNA006	^68^Ga-NOTA/NODAGA	hCD8 transfected adenocarcinoma cell line, Lung cancer, solid tumors	Preclinical Clinical Phase I	[27] [28] NCT05126927
	Human	VHH5v2	^18^F	T-cell Leukemia cell lines	Preclinical	[82]
	Human/Monkey	hCD8β Nb	^99m^Tc, ^68^Ga-NOTA, ^64^Cu-NOTA	Adenocarcinoma cell line	Preclinical	[83]
CD4	Human	CD4-Nb1	^64^Cu-NODAGA	Leukemia cell line	Preclinical	[84]
MHC-II	Mouse	VHH7	^18^F, ^64^Cu-NOTA,	Melanoma cell line, Pancreatic cancer cell line	Preclinical	[86, 95]
	Mouse	DC8	^18^F	Melanoma cell line, Pancreatic cancer cell line	Preclinical	[95]
CD11b	Mouse	DC13	^18^F,^64^Cu-NOTA,^89^Zr	Adenocarcinoma cell line, Melanoma cell line	Preclinical	[81, 86, 96]
MMR	Mouse	Cl1	^99m^Tc, ^111^In	Mammary adenocarcinoma cell line, Lung cancer cell line	Preclinical	[88, 97]
	Human/Mouse	3.49	^99m^Tc, [^18^F]SFB,^68^Ga-NOTA	Solid tumors, NSCLC, head and neck cancer, Solid malignancies undergoing ICI, Lymphoma	Clinical Phase I/IIa	[21, 22, 89] NCT04168528 NCT05933239 NCT04758650
Sirpα	Mouse	Nb15	^99m^Tc	Glioblastoma cell line	Preclinical	[90]
	Human	S36 Nb	^64^Cu-NODAGA	Adenocarcinoma cell line	Preclinical	[91]

CTLA-4: cytotoxic T-lymphocyte-associated protein 4; ^64^Cu: copper-64; ^8^F: fluorine-18; [^18^F]SFB: succinimidyl-4-[^18^F]fluorobenzoate; ^68^Ga: gallium-68; ICI: immune checkpoint inhibitor; ^111^In: indium-111; LAG-3: Lymphocyte activated Gene-3; MHC-II: major histocompatibility complex II; MMR: macrophage mannose receptor; NODAGA: 1,4,7-triazacyclononane,1-glutaric acid-4,7-acetic acid, THP: tris(hydroxypyridinone); NOTA: 1,4,7-Triazacyclononane-1,4,7-triacetic acid; NSCLC: non-small cell lung cancer; PBMCs: peripheral blood mononuclear cells; PD-L1/2: programmed death-ligand 1/2; SIRPα: Signal regulatory protein α; ^99m^Tc: technetium-99m; TIGIT: T cell receptor with Ig and ITIM domain; ^89^Zr: zirconium-89.

**Figure 3.**
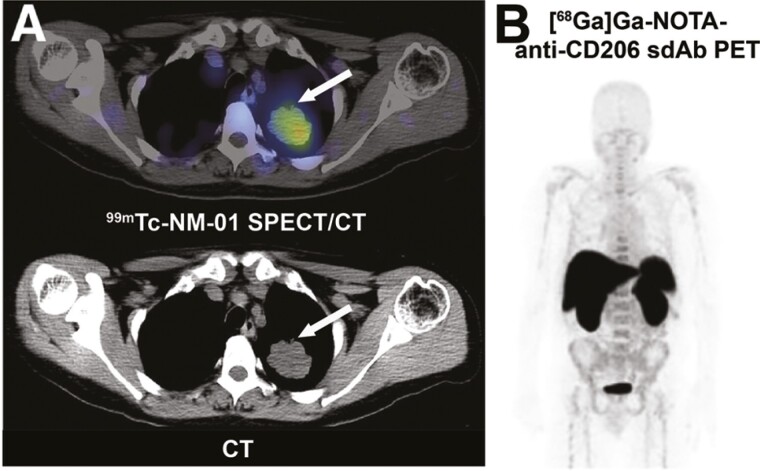
Examples of the clinical applicability of nanobody-based molecular imaging of programmed death ligand 1 and the macrophage mannose receptor (CD206). (A) Axial SPECT/CT (top) and corresponding CT-imaging (bottom) of the programmed death ligand 1 (PD-L1) targeting and ^99m^Tc-labeled nanobody NM-01 in a patient with a high PD-L1-expressing primary non-small cell lung cancer (arrow). (B) Maximum-intensity projection [^68^Ga]Ga-NOTA-anti-CD206 Nb (MMR3.49) images of a non-small cell lung cancer patient at 1.5 h post-injection. CT: computed tomography; ^68^Ga: gallium-68; PET: positron emission tomography; SPECT: single-photon emission computerized tomography; ^99m^Tc: technetium-99m. Images have previously been published in an adapted form by: (A) *JNM.* Xing *et al*. Early Phase I Study of a ^99m^Tc-Labeled Anti-Programmed Death Ligand-1 (PD-L1) Single-Domain Antibody in SPECT/CT Assessment of PD-L1 Expression in Non-Small Cell Lung Cancer. J Nucl Med. 2019;60(9):1213-1220. © SNMMI [65]; and (B) *JNM*. Gondry *et al*. Phase I Study of [^68^Ga]Ga-Anti-CD206-sdAb for PET/CT Assessment of Protumorigenic Macrophage Presence in Solid Tumors (MMR Phase I). J Nucl Med. 2023;64(9):1378-1384. © SNMMI [22], under a CC BY 4.0 license.

**Figure 4.**
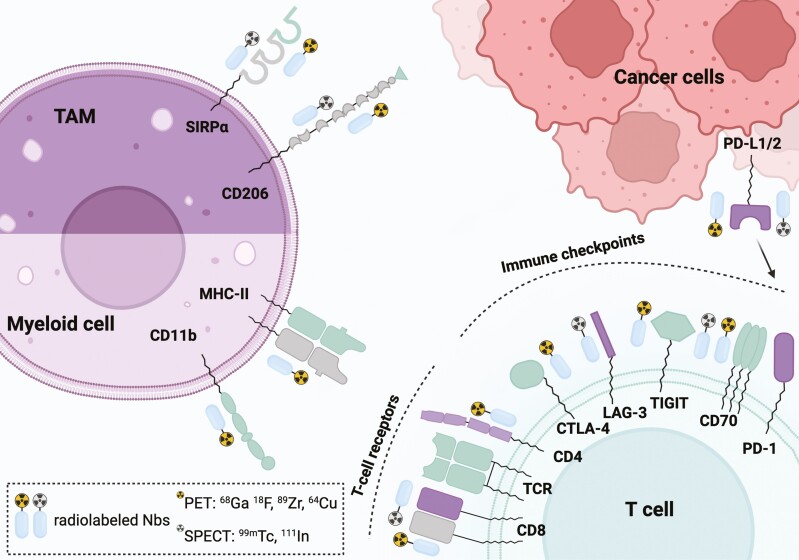
Overview of radiolabeled nanobodies targeting different receptors within the TME. Both markers of immune cell populations and immune checkpoint molecules are presented. CTLA-4: Cytotoxic T-lymphocyte associated protein 4; ^64^Cu: copper-64; ^18^F: fluorine-18; ^68^Ga: gallium-68; ^111^In: indium-111; LAG-3: Lymphocyte-activation gene 3; MHC-II: major histocompatibility complex II; MMR: macrophage mannose receptor; PD-1: Programmed cell death protein 1; PD-L1/2: Programmed death-ligand 1/2; PET: positron emission tomography; SIRPα: Signal regulatory protein α; SPECT: single-photon emission computerized tomography; TAM: tumor-associated macrophage; ^99m^Tc: technetium-99m; TCR: T-cell receptor; TIGIT: T cell immunoreceptor with immunoglobulin and ITIM domain; ^89^Zr: zirconium-89. Created with BioRender.com.

### CTLA-4

CTLA-4 belongs to the CD28 immunoglobulin subfamily, mainly expressed on regulatory T-cells (Treg) and activated T cells. It modulates T-cell activation and proliferation by competing with the immune-activating receptor CD28 for binding to the costimulatory ligands B7-1 and B7-2 [52]. The first-generation ICI, ipilimumab, was approved by the FDA in 2011 to treat advanced-stage melanoma, demonstrating increased overall survival. Yet, only a subset of patients exhibits a positive response, with over 80% of patients experiencing drug-related AEs [53]. Therefore, selecting patients who most likely benefit from anti-CTLA-4 therapy is crucial. Multiple studies have already reported on mAb- or antibody derivative-based diagnostic imaging of CTLA-4 [53]. However, only a few anti-CTLA-4 Nbs have been described. The majority are primarily explored for their therapeutic potential. Only one mCTLA-4-specific Nb (H11) was studied for *in vivo* PET imaging in syngeneic mouse tumor models. PET/CT imaging showed specific tracer accumulation within the TME of B16-F10 melanoma tumor-bearing mice at 90 min and 24 h post-injection with the ^18^F-labeled or ^89^Zr-labeled Nb H11, respectively [26].

### PD-L1/2

The interaction of PD-L1 and PD-L2 with PD-1 induces immune tolerance and promotes tumor escape. While predominantly expressed on tumor cells, PD-L1 is also found on macrophages, activated T cells, B cells, and brown adipose tissue [54]. Three mAbs against PD-L1 have already gained FDA approval: avelumab, atezolizumab, and durvalumab. Many studies have confirmed the correlation between PD-L1 expression and the prognosis of anti-PD-(L)1 therapy [55].

Several researchers have developed Nbs against mPD-L1 and hPD-L1 for noninvasive imaging. One research group demonstrated high target-to-background ratios in PD-L1-overexpressing tumor-bearing mice with multiple mPD-L1 Nbs (C3, C7, E2, and E4) and one hPD-L1 Nb (K2) following ^99m^Tc-labeling via SPECT/CT imaging. These tracers could distinguish between PD-L1-high and -low expressing tumors already at 1 h post-injection [56]. Additionally, the ^99m^Tc-labeled Nb C7 was used to visualize the spatiotemporal expression of mPD-L1 in mRNA-vaccinated B16 tumor-bearing mice, showing significant PD-L1-upregulation as early as one day post-vaccination, suggesting a potential synergy between cancer vaccination and PD-L1-blockade [30]. Finally, Nb K2 was evaluated as a PET tracer upon ^68^Ga-labeling. The ^68^Ga-labeled K2 demonstrated excellent *in vivo* targeting capabilities similar to its SPECT equivalent in melanoma and breast tumors with low kidney retention [33, 57, 58]. Another group also developed a ^99m^Tc-labeled mPD-L1 tracer (Nb MY1523), showing potential for evaluating dynamic PD-L1 expression *in vivo*, and its ability to follow-up therapy responses [59].

Several other PD-L1-specific Nb-based PET tracers have been reported. For instance, mPD-L1-specific Nbs B3 and A12, labeled with ^18^F and ^64^Cu, showed specific accumulation in brown adipose tissue, indicating a metabolic role for PD-L1 [60]. Additionally, ^68^Ga-labeled anti-hPD-L1 Nbs Nb109 and APN09 demonstrated effective PD-L1-targeting in various tumor models and patient-derived lung xenografts. These Nbs enable specific detection of endogenous PD-L1 and dynamic changes in its expression, offering the potential for personalized treatment regimens and prognostic efficacy assessment [61–64]. Following positive preclinical results, ^68^Ga-labeled Nb APN09 entered a phase I clinical trial (NCT05156515), where PET/CT imaging with [^68^Ga]Ga-THP-APN09 in nine NSCLC patients showed a positive correlation between PD-L1 expression and tracer accumulation in tumors [64]. ^99m^Tc-NM-01 is another Nb-based PD-L1 tracer being clinically evaluated for SPECT/CT imaging in NSCLC patients (NCT02978196). Clinical results show no drug-related AEs, adequate dosimetry, and tracer accumulation at the tumor site correlating with PD-L1 expression (Fig. 3A) [65]. KN035, a PD-L1 Nb showing therapeutic effect was also explored for its diagnostic potential. ^89^Zr-labeled KN035 was evaluated in patients with PD-L1-positive solid tumors, showing the detection of metastatic foci and the ability to monitor and predict the site of adverse reactions in antitumor immunotherapy. This further demonstrated the theranostic potential of this Nb [66]. Recently, some other PD-L1-specific Nbs have entered clinical testing as well. These include the [^68^Ga]Ga-Nb-1 for PET/CT imaging of patients with solid tumors (NCT06383598), and the [^68^Ga]Ga-NOTA-RW102 along with its derivative for PET/CT imaging in NSCLC patients (NCT06165874) [67].

In addition to PD-L1, PD-L2, a less extensively studied ligand of PD-1, is also under clinical investigation. A study is ongoing to target PD-L2 in patients diagnosed with various solid malignancies undergoing surgery or biopsy, using the ^68^Ga-labeled Nb Mirc415, and results are awaited (NCT05803746).

### Next-generation immune checkpoints

LAG-3 is an example of a next-generation IC exhibiting high expression, for instance in exhausted T cells [68]. To date, a few Nb-based tracers have been developed specifically for h/mLAG-3. Upon ^99m^Tc-labeling, anti-hLAG-3 Nb 3187 and anti-mLAG-3 Nbs 3206 and 3132 demonstrated specific tumor uptake in LAG-3-overexpressing tumor-bearing mice. Additionally, tracer accumulation was detected in peripheral organs containing LAG-3-expressing cells, such as spleen and lymph nodes [69, 70]. Furthermore, the mLAG-3-specific Nb 3132 was able to quantify dynamic LAG-3 expression within the TME and tumor-draining lymph nodes of PD-1-treated MC38 tumor-bearing mice, revealing a compensatory LAG-3 upregulation as a consequence of PD-1 blockade [71].

Another IC discovered in 2009 is TIGIT. The TIGIT pathway is known to mediate both innate and adaptive immune responses and contribute to effector cell exhaustion [72]. Although numerous clinical trials are investigating TIGIT-targeting therapies, current findings indicate varying efficacy, necessitating further evaluation of the significance of TIGIT immunotherapies [73]. Currently, TIGIT diagnostic tracers are still in preclinical development. Recently, two ^99m^Tc-labeled Nb-based SPECT tracers (anti-mTIGIT Nb 16988 and anti-hTIGIT Nb 16925) were developed. These ^99m^Tc-labeled Nbs showed specific uptake in TIGIT-expressing TC-1 tumors at 1 h post-injection, serving as a proof-of-concept for future applications [74]. Another study reported a ^68^Ga-labeled Nb-based hTIGIT tracer (Nb138) for PET imaging, showing specific uptake from 0.5 h up to 2 h post-injection in A375 melanoma-bearing nude mice [75]. With continuous research on novel ICs, many other Nbs targeting ICs will likely emerge in the near future.

In addition to the inhibitory ICs, the costimulatory receptor CD70 has emerged as an attractive target for immunotherapies. CD70 overexpression has been observed in various solid and hematological malignancies, resulting in immune evasion and tumor progression [76]. Several mAbs and drug-conjugates have already been developed and are currently undergoing clinical evaluation. To allow CD70 visualization, ^68^Ga-labeled NOTA-conjugated anti-hCD70 Nb demonstrated specific uptake in a CD70^high^ renal carcinoma model [76]. Recently, a first-in-human study has been reported with ^18^F- labeled CD70-specific Nb RCCB6 for PET/CT imaging of patients with clear cell renal cell carcinoma [77].

### Nanobody-based imaging of immune cell populations

Efforts have also been made to track immune cell dynamics to predict or follow-up immunotherapy response. Several Nbs against markers specific for T-cell populations, including CD8^+^ cytotoxic T cells (CTLs) and CD4^+^ T-helper (Th) cells, as well as myeloid cells, including macrophages, have been evaluated as diagnostic tracers for this purpose (Table 3 and Fig. 4).

### T-cell imaging

Numerous T-cell-based immunotherapies have found their way to the clinic [78]. Moreover, numerous studies have linked the presence of T-cell populations, specifically CTLs, in the TME with enhanced therapy responses [79]. Therefore, noninvasive tracking of T-cell populations has gained more interest over time. To date, four anti-CD8 Nb-based tracers were developed and tested (pre-)clinically. The first reported Nb visualizing mCTLs (VHH-X118) was described in 2017, showing a sub-nanomolar affinity for mCD8α [80]. Upon PEGylation and ^89^Zr-labeling, VHH-X118 could visualize intratumoral CTLs in a B16 melanoma and a Panc02 pancreatic tumor at 24 h and 48 h post-injection. Furthermore, CTL dynamics and intratumoral biodistribution allowed us to predict therapy response, revealing a correlation between a more uniform distribution of CTLs and a favorable therapy response [81]. More recently, the ^68^Ga-labeled anti-hCD8α tracer SNA006 showed specific and rapid accumulation in hCD8-overexpressing MC38 tumor-bearing mice already at 0.5 h post-injection [27, 28]. To facilitate further clinical translation of ^68^Ga-labeled SNA006, two doses (25 µg/kg and 150 µg/kg) were tested in non-human primates to monitor the tracer’s safety and biodistribution at multiple timepoints up to 4 h post-injection. Interestingly, the lowest dose resulted in the highest uptake in CD8-rich organs such as the spleen, bone marrow, and lymph nodes. Additionally, preliminary results from a phase I study (NCT05126927) showed a linear correlation between tracer uptake and CD8 expression. While these initial clinical findings are promising, larger cohort studies are required to establish significant correlations between CD8 expression and tracer uptake to use the tracer for therapy prediction [28].

A third ^18^F-labeled anti-hCD8α tracer (VHH5v2) was recently developed, allowing rapid detection of differences in endogenous CD8 expression across three xenograft models. Notable, the tracer visualized both intermediate and low CD8-expressing tumors as early as 10 min post-injection and remained stable throughout the 1-h imaging experiment [82].

All the previous PET tracers target the α-chain, which can form CD8α homodimers on cells other than T cells. In a recent study, a Nb against the β-chain of the hCD8 protein was developed, showing more specific targeting of CTLs compared to the anti-hCD8α Nbs. Upon ^99m^Tc- or ^68^Ga-labeling, this anti-hCD8β Nb showed specific targeting of CTLs (1 h post-injection) in CD8-rich organs and MC38 tumors in hCD8 transgenic mice. Furthermore, the Nb demonstrated its capability to visualize T-cell dynamics over time in the same mouse model. Its prognostic value was further confirmed by correlating intratumoral CTLs at early timepoints and tumor growth over time. Finally, a proof-of-concept study in non-human primates showed a good biodistribution profile at 1 h after administration of 500 µg of ^64^Cu-Nb [83].

Besides CD8^+^ T cells, CD4^+^ Th cells also play an essential role in initiating immune responses. To date, one ^64^Cu-labeled Nb targeting hCD4^+^ Th cells has been described with optimal signal-to-background ratios 3 h post-injection in various CD4-rich organs in hCD4 knock-in mice [84]. However, the marker CD4 has also been found on other cell types besides CD4^+^ Th cells (Tregs, peripheral monocytes, and other antigen-presenting cells). Therefore, to specifically target CD4^+^ Th cells, more specific markers in addition to CD4 are warranted [84].

### Myeloid cells

A dual role in the TME can be attributed to myeloid lineage cells, consisting of monocytes, macrophages, dendritic cells, and granulocytes, identified by cell surface markers CD11b or MHC class II [85]. These myeloid cells can either aid tumor cells in maintaining tissue homeostasis and modulating T-cell responses or engage in direct or indirect tumor cell elimination by activating CTLs [85]. Consequently, two anti-mMHC-II Nbs (VHH7 and DC8) and one anti-mCD11b Nb clone (DC13) were generated to visualize myeloid cells. Specific binding of the ^18^F-labeled Nbs (VHH7 and DC13) to MHC-II^+^ cells and CD11b^+^ cells in both early and late-stage melanoma 1.5 h post-injection was reported [86]. Furthermore, PET/CT imaging with a ^89^Zr-PEGylated-DC13 tracer version allowed the stratification of PD-1 responders from non-responders, with responders showing a more uniform tracer distribution within the tumor than non-responders [81].

### Tumor-associated macrophages (TAMs)

TAMs display a diverse spectrum of markers indicative of either anti- or pro-tumoral phenotypes, with the latter correlating with poor prognosis across various cancer types [87]. A well-studied pro-tumoral TAM marker is the macrophage mannose receptor (MMR), also known as CD206. The first reported anti-mMMR Nb (Cl1), after being labeled with ^99m^Tc and ^111^In, showed macrophage-specific uptake in mouse breast and lung tumors. A high accumulation was also observed in macrophage-rich organs such as the spleen and liver. Co-administration of an excess of unlabeled bivalent anti-mMMR Nb effectively blocked the extra-tumoral binding sites and significantly increased the tumor-to-blood ratio [88]. For clinical translation, a m/h cross-reactive clone (MMR3.49) with comparable binding affinity and tumor-targeting potential was generated and characterized. Recently, a phase I study performed with the ^68^Ga-labeled MMR3.49 showed overall low total tumor uptake levels in six patients. However, patients with the highest uptake later developed progressive disease, suggesting a predictive value for the tracer (Fig. 3B) [22]. Currently, two phase II studies are ongoing in patients with different malignancies (NCT05933239 & NCT04758650) [21, 22, 89].

Along the same line, the myeloid-specific IC signal regulatory protein alpha (SIRPα) was shown to be expressed on tumor oligodendrocytes, type 2 conventional DCs, monocytes, and TAMs. In 2021, imaging of SIRPα-expressing cells in an intracranial glioblastoma mouse model using a ^99m^Tc-labeled anti-mSIRPα Nb (Nb15) was shown even without additional permeabilization of the blood–brain barrier. A similar result, as seen with the MMR Nb was observed with accumulation in the tumor, as well as in the liver and spleen [90]. More recently, the potential of an anti-hSIRPα Nb was assessed in an immunocompetent mouse model. MC38 adenocarcinoma cells overexpressing hCD47 (MC38-hCD47^+^) were inoculated into hSIRPα/hCD47 knock-in mice and wild-type mice. The ^64^Cu-Nb showed rapid accumulation within the tumor of the hSIRPα/hCD47 knock-in mice via PET/MRI shortly after injection (6 min) and remained stable for 6 h. A 3-fold higher uptake in the MC38-hCD47^+^ tumor of hSIRPα/hCD47KI mice compared to wild-type mice was shown 3 h post-injection [91]. These Nbs could help improve or predict macrophage-targeting therapies employing SIRPα in the future.

## Discussion

Current conventional approaches to monitor treatment response and tumor progression include immunohistochemistry and ^18^F-2-fluoro-2-deoxyglucose ([^18^F]FDG)-PET. [^18^F]FDG-PET is the most widely used imaging method in clinical practice. However, FDG only monitors alterations in glucose metabolism (i.e. malignant cells) but does not provide information on the molecular characteristics of the tumor. Furthermore, immune cell activation, due to immunotherapy response or inflammation, will also be detected with [^18^F]FDG-PET, resulting in false-positive results [98]. In contrast, lesions with lower metabolic activity will not be detected via [^18^F]FDG-PET [98]. Due to these inherent limitations, research has focused on the development of more tumor-specific imaging agents. Multiple tracers have been developed with several ones being clinically tested or even approved. A key example is the FDA-approved PSMA-targeting tracer, [^68^Ga]Ga-PSMA-11, for imaging of metastatic castration-resistant prostate cancer, showing a higher prognostic value compared to [^18^F]FDG-PET to stratify patients for PSMA-targeted therapy [99].

In contrast to tumor-specific antigen imaging, imaging of the TME is still in an earlier research phase, with only a few tracers being clinically tested. However, imaging of immunotherapy responses may hold great prognostic or predictive value. To date, imaging of ICIs or general immune cell populations for patient stratification, response monitoring, and drug resistance prediction has been the main focus. Here, Nb-based tracers have shown their added value. It seems logical that in the future, more novel Nb-based tracers will be developed against newly discovered ICIs and other immune cell populations (e.g. mast cells, neutrophils, basophils, and eosinophils). In addition, some of the current immune cell markers used (e.g. CD8α, MMR, and SIRPα) are not exclusively expressed on specific immune cell subsets, which may hamper their diagnostic potential. Also here, the development of novel and more specific (Nb-based) immunotracers against these immune subsets could be beneficial and result in enhanced prognostic or predictive potential.

Recent advances in spatial omics technologies have shown the dynamic nature of the TME and the importance of the interplay between different immune cell populations in the TME. This complex and dynamic environment suggests that a single tracer may not be sufficient to predict or follow-up immunotherapy responses. To this end, multiplex imaging, by combining different PET and/or SPECT tracers, could give more insight into the TME. Here, Nb-based tracers could be essential due to their inherent short *in vivo* half-life, resulting in the ability to perform multiple scans in a short timeframe. Multiple options are possible, including subsequent imaging of two PET tracers, one PET and one SPECT Nb-based tracer, or simultaneous imaging of two SPECT Nb-based tracers. While nuclear multiplex imaging is still in its infancy, it is not unlikely that this approach may hold great value in the immune-imaging field.

Employing nuclear imaging to monitor the spatiotemporal changes of TME-specific markers and predict immunotherapy response has gained interest in recent years. Currently, only a few imaging agents have been tested (pre-)clinically and shown predictive value during ICI, including tracers against CD69 (an immune activation marker) [100], granzyme B (a downstream effector of CTLs) [101], and CD8α [81]. Beyond monitoring ICI response, it would also be interesting to monitor other immunotherapies with Nb-based immunotracers, such as CAR-T cell therapy and cancer vaccines. CAR-T cell therapy is one of the most promising immunotherapies, showing remarkable results in hematological cancers, though its efficacy in solid tumors is still limited [102]. Currently, bioluminescent and PET/CT imaging are being exploited to track CAR-T-cell distribution, aiding in the interpretation of therapy responses and limitations. Nb-based tracers targeting CD8, T-cell activation, and exhaustion markers enable multiple timepoint imaging with short-lived radionuclides for PET/CT imaging. This approach paves the way for tracking CAR-T-cell dynamics, infiltration, and accumulation over time. Furthermore, these insights could support the development of effective strategies for treating solid tumors with CAR-T cell therapy.

Another promising immunotherapy is the delivery of tumor-specific antigens to the patient’s body through cancer vaccines to elicit an immune response. Although eight of them have been approved by the FDA, none have demonstrated a significant clinical impact yet [103]. Nbs can be employed to either investigate the migration route of these tumor-specific antigens to the tumor or visualize the response of antigen-specific CTLs. Overall, Nb-based imaging of cancer vaccines could potentially provide insights into the immune responses evoked by the tumor-specific antigens, thereby aiding in the design of better vaccines.

To date, multiple groups have been pursuing the clinical translation of Nb-based tracers. This is quite remarkable since diagnostic tracers are less economically interesting compared to therapeutic Nbs. However, this is most likely due to less strict clinical trial requirements including single-(micro)dosing and overall lower costs. Furthermore, the clinical translation of Nb-based tracers compared to mAb-based tracers offers some additional benefits, mainly due to their smaller size. This includes the use of short-living isotopes, resulting in reduced patient radiation burden and the possibility of same-day imaging. Furthermore, the ability to use bacteria or yeast as GMP production vectors, compared to mammalian cells for mAb-based tracers, and the need for lower quantities of product lowers the overall cost as well. However, the non-human origin of Nbs must be considered with respect to immunogenicity even with the fact that Nbs are generally considered low-immunogenic [20]. As such, a thorough immunogenicity assessment for each Nb is required and humanization of Nbs has been proposed as a strategy to facilitate clinical translation of Nb-based tracers [23].

To conclude, nuclear imaging offers a unique window into the complex interplay between tumors and the immune system, providing valuable insights to guide treatment decisions and explore new targets for future immunotherapies. Nbs hold promise as versatile nuclear imaging agents, demonstrating their value in (radio)theranostics and their efficacy in monitoring immunotherapy responses. This could help address future clinical questions in the field of immune-imaging.

## Abbreviations

α: Alpha decay
ADAs: Anti-drug antibodies
ADCs: Antibody-drug conjugates
AEs: Adverse events
β^+^: Beta plus decay
β-: Beta minus decay
CEA: Carcinoembryonic antigen
CLDN18.2: Claudin18.2
CTLs: CD8^+^ cytotoxic T cells
CTLA-4: Cytotoxic T-lymphocyte-associated protein 4
^64^Cu: copper-64
DCs: Dendritic cells
DFO: Desferrioxamine
DOTA: 1,4,7,10-tetraazacyclododecane-1,4,7,10-tetraacetic acid
DTPA: Diethylenetriaminepentaacetic acid
ε: Electron capture
ECM: Extracellular matrix
^18^F: Fluorine-18
Fab: Antigen-binding fragment
FAP: Fibroblast activation protein
[^18^F]AlF: Aluminum-[^18^F]fluoride
Fpy-TFP: 6-[^18^F]fluoronicotinyl-2,3,5,6-tetrafluorophenyl ester
[^18^F]SFB: N-succinimidyl-4-[^18^F]fluorobenzoate
^68^Ga: Gallium-68
HER2: Human epidermal growth factor 2
ICs: Immune checkpoints
ICIs: Immune checkpoint inhibitors
^111^In: Indium-111
^131^I: Iodine-131
IT: Isomeric transition
LAG-3: Lymphocyte-Activated Gene-3
mAbs: Monoclonal antibodies
MHC-II: Major histocompatibility complex II
MMR: Macrophage mannose receptor
MW: Molecular weight
Nbs: Nanobodies
NODAGA: 1,4,7-triazacyclononane,1-glutaric acid-4,7-acetic acid
NOTA: 1,4,7-Triazacyclononane-1,4,7-triacetic acid
NSCLC: Non-small-cell lung carcinoma
PBMCs: Peripheral blood mononuclear cells
PD-1: Programmed death-1
PD-L1/2: Programmed death-ligand 1/2
PEG: Polyethylene glycol
PET: Positron emission tomography
PSMA: Prostate-specific membrane antigen
ScFv: Single-chain variable fragment
sdAb: Single domain antibody
SGMIB: N-succinimidyl 4-guanidino-methyl-3-iodobenzoate
SIRPα: Signal regulatory protein alpha
SPECT: Single photon emission computed tomography
TAM: Tumor-associated macrophage
^99m^Tc: Technetium-99m
TCR: T-cell receptor
Th cells: CD4^+^ T-helper cells
THP: Tris(hydroxypyridinone
TIGIT: T cell receptor with Ig and ITIM domain
TIM-3: Mucin domain-containing protein 3
TME: Tumor microenvironment
Tregs: Regulatory T cells
Trop2: Tumor-associated calcium signal transducer 2
TRT: Targeted-radionuclide therapies
^89^Zr: Zirconium-89
